# Acaricidal Activity and Field Efficacy Analysis of the Potential Biocontrol Agent *Bacillus vallismortis* NBIF-001 against Spider Mites

**DOI:** 10.3390/microorganisms10091750

**Published:** 2022-08-30

**Authors:** Lei Zhu, Ling Chen, Yong Min, Fang Liu, Xianqing Liao, Ben Rao, Yimin Qiu, Wei Chen, Kaimei Wang, Ziwen Yang, Ronghua Zhou, Yan Gong, Xiaoyan Liu

**Affiliations:** 1 National Biopesticide Engineering Technology Research Center, Wuhan 430064, China; 2 Hubei Biopesticide Engineering Research Center, Hubei Academy of Agricultural Sciences, Wuhan 430064, China; 3 Key Laboratory of Microbial Pesticides, Ministry of Agriculture and Rural Affairs, Wuhan 430064, China; 4 Hubei Hongshan Laboratory, Wuhan 430070, China

**Keywords:** spider mites, *Bacillus vallismortis*, acaricidal activity, field efficacy, protein BVP8, biocontrol

## Abstract

In recent years, spider mites have caused considerable economic losses to global agriculture. However, currently available management strategies are limited because of the rapid development of resistance. In this study, *Bacillus vallismortis* NBIF-001 was isolated and evaluated for its acaricidal activity. NBIF-001 exhibited a significant lethal effect on spider mites within 48 h. The median lethal concentration (LC_50_) of the culture powders (3.2 × 10^10^ CFU/g) was 50.2 µg/mL for *Tetranychus urticae* (red form), 18.0 µg/mL for *T. urticae* (green form), and 15.7 µg/mL for *Panonychus citri* (McGregor). Cultivation optimisation experiments showed that when the number of spores increased, fermentation toxicity also increased. Moreover, field experiments demonstrated that NBIF-001 performed well in the biocontrol of *P. citri*, which showed a similar corrected field efficacy with the chemical control (67.1 ± 7.9% and 71.1 ± 6.4% after 14 days). Genomics analysis showed that NBIF-001 contains 231 factors and seven gene clusters of metabolites that may be involved in its acaricidal activity. Further bioassays of the fermentation supernatants showed that 50× dilution treatments killed 72.5 ± 5.4% of the mites in 48 h, which was similar with those of the broth. Bioassays of the supernatant proteins confirmed that various proteins exhibited acaricidal activity. Five candidate proteins were expressed and purified successfully. The bioassays showed that the small protein BVP8 exhibited significant acaricidal activity with an LC_50_ of 12.4 μg/mL (*T. urticae*). Overall, these findings suggest that *B. vallismortis* NBIF-001 is a potential biocontrol agent for spider mite management.

## 1. Introduction

Phytophagous mites are serious pests in various agricultural crops worldwide, causing over $4500 of damage per hectare by direct feeding, or in Eriophyoidea, by transmitting plant pathogens and viruses as well [1,2]. China is one of the world’s largest agricultural producers, with many crops and agroecosystems in diverse and heterogeneous climatic zones. With the rapid development of agriculture, the damage caused by spider mites has attracted attention in recent years. For example, the two-spotted spider mite (*Tetranychus urticae* Koch, also known as *T. cinnabarinus* (Boisduval)) caused over 50–70% yield loss in cassava (*Manihot esculenta* Crantz) production [3]. Orchard workers commonly apply pesticides more than 20 times a year, with up to 50% of sprays being applied to reduce the citrus red mite (*Panonychus citri* (McGregor)) [4].

Chemically synthesised acaricides are the main tools used to control spider mites. According to the China Pesticide Information Network (http://www.chinapesticide.org.cn/), as of 1 April 2022, there are 1121 registered acaricides. The top three acaricides are propargite (13.7%, proportion of registered cases), pyridaben (10.7%), and azocyclotin (9.8%). Abamectin is the fourth most common biological source (5.4%). It is usually used as a compound acaricide with chemicals, such as abamectin + pyridaben (4.0%) and abamectin + propargite (1.1%). The total proportion of the various registered compound acaricides is 15.1%, of which almost all contains abamectin. However, the small body size and rapid reproductive cycle of spider mites allow them to easily generate resistance in a short period of time [5,6]. Along with increasing legal restrictions on pesticides [7], agricultural insect and mite pest control has been driven to apply more non-chemical or biological solutions. Mass-produced spider mite natural enemies have been used for biological pest control for decades, but their use is subject to release date, temperature, and other environmental factors [8,9]. Applying mycoinsecticides, including *Beauveria bassiana* and *Metarhizium anisopliae*, is another effective means of environmentally friendly insect control [10], and previous studies have assessed their potential commercial application for controlling spider mites [11,12]. In addition, mineral oil is used for initial spider mite control given its compatibility with predatory mite application [13]. Essential oils from diverse plants have repellent or acaricidal effects against the two-spotted spider mite, *Tetranychus urticae* under laboratory conditions [2]. Compared with the 20-fold insecticides registered in China (18,626 products), it is important to find more acaricidal products that can cope with serious threats caused by increasing resistance.

*Bacillus* strains are distributed worldwide and have been used as commercial insecticides, bactericides, and plant growth-promoting agents for several decades [14,15]. The main targets of insecticides and nematicides are the larvae of Lepidoptera, Diptera, Coleoptera [16], and nematodes [17]. Given the special feeding modes, sucking pests may not directly ingest bacterial cells or their toxins from the microbial pesticides sprayed on the plants and would not be killed or weakened as insects in the action mode of *Bacillus* insecticides [16]. However, various *B. thuringiensis* strains have been found to kill root-knot nematodes which also use a similar sucking way on plant root [18]. Interestingly, *Bacillus velezensis* W1 has been found to exhibit acaricidal activity, and cyclodipeptides, bacillomycins, macrolactins, and surfactins have been identified from broth supernatants and may have acaricidal potential, as demonstrated by bioassays [19] and virtual predictions [20]. Through sophisticated fermentation technology, *Bacillus* may be a potential resource for developing high-efficacy and low-cost acaricides.

In this study, thousands of *Bacillus* strains that have been isolated over the past 20 years in our laboratory were screened for acaricidal activity against *T. urticae* (red and green form) and *P. citri* as targets. NBIF-001, which was the first to show excellent acaricidal activity in our laboratory, was characterised and confirmed by indoor bioassays and field experiments. Genomics analyses, compound identification, and bioassays of purified proteins indicated that various proteins in fermentation supernatants, especially the small protein BVP8, play important roles in toxicity. The results of the field experiments suggest that NBIF-001 has great potential for spider mite control.

## 2. Materials and Methods

### 2.1. Biological Materials

The strains, plasmids, and primers used in this study are listed in Table S1. *B. vallismortis* NBIF-001 (CCTCC M 2015087) was isolated from soil collected in 2010 from Shangri-La, China [21]. *B. thuringiensis* subsp. *kurstaki* HD-1 (BGSC no. 4D1), the primary U.S. reference standard for commercial *B. thuringiensis* products globally, was stored in our laboratory and used as a control. The *Bacillus* strains were inoculated and grown in Luria–Bertani (LB) medium at 30 °C and shaken at 200 rpm. When the spores had been freed (≥90%) of germinated spores, growing or sporulating cells, and cellular debris, the culture was collected by centrifugation at 4000 rpm for 10 min, lyophilised, and stored at 4 °C until further use. *Escherichia coli* strains were cultured at 37 °C and shaken at 250 rpm in LB medium containing 50 ng/μL of kanamycin to maintain the plasmids for protein expression.

The *T. urticae* (red and green form) used for the bioassays were raised on cowpea seedlings (*Vigna unguiculata*) in an insectary for over five years without pesticide exposure. The insectary conditions included 25 ± 1 °C and 75 ± 5% relative humidity with a 14-h light/10-h dark cycle. *P. citri* were collected from kumquat leaves in greenhouses at the Institute of Fruit and Tea, Hubei Academy of Agricultural Science, China.

### 2.2. Acaricidal Assays

Preliminary bioassays were performed using the slide-dip method recommended by the Chinese Agricultural Standard [22]. In brief, double-sided adhesive tapes (2 cm in length) were attached to glass slides. Approximately 30 active adult mites were selected, and their backs were attached to the adhesive tape on each slide. Four replicates for each individual treatment. The slides were dipped into the various treatments mentioned below and gently swung for 5 s. Filter paper was used to remove the residue. The slides were then placed in a transparent container and kept in a growth chamber (conditions set to match the insectary described above) for 48 h. The mites were observed under a stereoscopic microscope (Olympus SZ61). Those that exhibited immobility or irregularly trembling legs were considered dead. If the mortality in the control group exceeded 20%, then the bioassay was nullified and repeated. Mortality was corrected by applying the formula in the Chinese Agricultural Standard [22]:

Corrected mortality = (test mortality% − control mortality%)/(100 − control mortality%) × 100%(1)

In the bioassays of the NBIF-001 culture, seven concentrations (0.1, 1.0, 10.0, 25.0, 50.0, 100.0, and 200.0 µg/mL in 0.05% Triton X-100, as 3.2 × 10^4^ to 6.4 × 10^6^ CFU/mL, respectively) were used in four replicates in different assays. The negative controls were 200.0 µg/mL HD-1 and 0.05% Triton X-100. In the bioassays of the fermentation broth, 1×, 10×, 50×, 100×, 500×, and 1000× dilutions of fermentation broth, supernatants were filtered through a 0.22-μm filter, and cell resuspensions were used. To prepare the cell resuspension, the cells were resuspended in phosphate-buffered saline (PBS) buffer (pH 7.4) at the same volume as the broth and supernatants in the same diluted treatment. Fermentation medium was used as a negative control. The gradient concentrations of the fractions of proteins from ammonium sulphate precipitation or purified proteins were used in the bioassays. PBS (pH 7.4) and bovine serum albumin (BSA, 1000.0 μg/mL) were used as negative controls. The positive controls for the bioassays on *T. urticae* were avermectins (1.8% emulsifiable concentrate, EC, Hansencn, pesticide registration number PD20090654, China) and cyetpyrafen (30% suspension concentrate, SC, Baozhuo, pesticide registration number PD20181622, China) at 1000× and 2000× dilutions according to the manufacturer’s instructions (×indicates the dilution ratio). The final working concentrations were 18.0 and 150.0 µg/mL, respectively.

### 2.3. Optimisation of NBIF-001 Cultivation Conditions

For the growth kinetics, the freshly inoculated cultures were incubated in LB medium at 30 °C and shaken at 200 rpm. Optical densities were measured every 4 h for 48 h at 600 nm using a spectrophotometer. The seed liquid for fermentation optimisation experiments was obtained at 16 h by the growth kinetics experiment, which is the end of logarithmic growth with highest cell number and maintained activity (Figure S1a). The initial fermentation medium was used as described above. For the preliminary optimisation of carbon and nitrogen sources, four other carbon sources (glucose, glycerol, sucrose, and corn flour) and eight nitrogen sources (urea, fish meal, industrial peptone, yeast extract, silkworm pupae meal, peanut meal, sesame meal, and corn steep liquor) were used at the same dose as corn starch and soybean meal. Based on the single-factor experiments, a four-factor, nine-level orthogonal experiment was designed. The number of spores presented in the fermentation broth was used as the inspection index; corn flour was the carbon source; soybean meal, yeast extract, and corn steep liquor were the nitrogen sources; and the inorganic salt content was maintained with 1.0 g/L of calcium carbonate, 0.3 g/L of magnesium sulphate, and 0.1 g/L of potassium dihydrogen phosphate. The fermentation conditions were optimised for pH, culture temperature, inoculum concentration, and rotation speed. The pH of the optimised medium was adjusted to 6.0, 6.5, 7.0, 7.5, and 8.0. The cultures were grown at 25, 30, 35, 40, and 45 °C. Inoculation concentrations of 1, 3, 5, 7, and 10% were tested. The rotation speeds were set to 120, 150, 180, 200, and 240 rpm. Optimal parameters were determined by counting the number of cells and spores in the fermentation liquor.

### 2.4. Field Efficacy Trials of NBIF-001 Agents on Citrus

The experiment was conducted in a field of a citrus orchard in June 2021 in Yangxin, Hubei, China, with an area of approximately 1.3 ha. *Citrus reticulata* (Ai Yuan 38) trees were 1.5–2 m high and occupied approximately 5–10 m^2^. The meteorological conditions on the day of treatment and 14 days after treatment are shown in Table S2. The spraying time ranged from 8:00 to 10:00 AM, depending on the wetness or dryness of the morning dew on the surfaces of the leaves and intensity of sunlight. Based on the Guidelines for the Field Efficacy Trial-Acaricides Against Spider Mites on Citrus [23], the treatments included 50×, 100×, and 200× dilutions of NBIF-001 wettable powder, a 2000× dilution of cyetpyrafen 30% SC, and water control. The wettable powder of NBIF-001 was prepared by mixing one part of raw NBIF-001 fermentation powder with four parts of diatomaceous earth and 0.5 parts of ammonium sulphate (for wettability). Before the treatment, the total numbers of mites in the selected fields were 199 ± 7 (water control), 223 ± 35 (50× dilution of NBIF-001 wettable powder with 1.0 × 10^10^ CFU/g), 195 ± 58 (100× dilution of NBIF-001), 165 ± 52 (200× dilution of NBIF-001), and 190 ± 131 (2000× dilution of cyetpyrafen 30% SC). A 20-L manual sprayer (type 3WBS-D-16B) with a working pressure of 0.45 Mpa and rated flow of 2–4 L/min was used. The leaves were entirely moist on both sides, but no liquid dripped from the leaves. Two citrus trees and four replicates were used for each treatment. The number of mites on five branches from five sides of the same tree was checked before treatment and at 1, 3, 7, and 14 days after treatment. The mite reduction rate and corrected field efficacy were calculated using the following equations:

Mites reduced rate = (the number before treatment − the number after treatment)/(the number before treatment) × 100%(2)

Corrected field efficacy = (mites reduced rate% − control mites reduced rate%)/(100 − control mites reduced rate%) × 100%(3)

### 2.5. Whole-Genome Sequence Analysis and Annotation

Genome sequencing and the assembly of *B. vallismortis* NBIF-001 have been described previously [21]. In the present study, putative acaricidal, colonisation, and other factors or features that may be associated with acaricidal activity were annotated and analysed. Functional annotation was performed by searching the KEGG [24], COG [25], and Virulence Factor [26] databases. A signal peptide analysis was performed using SignalP 6.0 [27]. The secondary metabolite biosynthesis gene clusters were identified using antiSMASH 6.0 bacterial version [28].

### 2.6. Crude Protein Extraction from the Fermentation Broth Supernatants of NBIF-001

The partial purification of the proteins from the fermentation broth supernatants of NBIF-001 was performed via ammonium sulphate precipitation and dialysis, according to a modified method [29]. NBIF-001 was inoculated and grown in fermentation medium at 30 °C and shaken at 200 rpm. The fermentation medium included 10.0 g of corn starch, 20.0 g of soybean meal, 1.0 g of calcium carbonate, 0.3 g of magnesium sulphate, and 0.1 g of potassium dihydrogen phosphate dissolved in 1.0 L of water (pH 7.5). After 24 h of fermentation, the supernatant was collected via centrifugation. Ammonium sulphate was slowly added to the supernatant until 30% saturation was reached. The mixture was stirred constantly at 4 °C for 6 h then centrifuged at 12,000× *g* for 20 min at 4 °C. The precipitates were re-dissolved in PBS (pH 7.4), and the supernatants were subjected to 50% ammonium sulphate saturation. This procedure was repeated until a precipitate with 90% ammonium sulphate saturation was obtained. The fractions were dialyzed (cut off 2000 Da) with PBS buffer (pH 7.4) three times at 4 °C for 24 h to completely remove the ammonium sulphate. The protein concentration of the dialysed supernatant was detected using a NanoDrop 2000 spectrophotometer (Thermo Fisher, Waltham, MA, USA).

### 2.7. Protein Mass Spectrometry

The proteins were digested with trypsin at 37 °C overnight. The peptides were extracted with 50% acetonitrile/2% formic acid, followed by 100% acetonitrile. The peptides were then dried and resuspended in 98% water/2% acetonitrile with 0.1% formic acid (mobile phase A). The tryptic peptides were separated by reverse-phase liquid chromatography using a homemade reversed-phase analytical column (75 μm × 15 cm) in an EASY-nLC 1000 UPLC system (Thermo Scientific, Waltham, MA, USA). The gradient comprised an increase from 8–35% mobile phase B (20% water/80% acetonitrile with 0.1% formic acid) over 60 min, 35–80% over 5 min, and then holding at 80% for the last 3 min, all at a constant flow rate of 0.3 μL/min. The peptides were subjected to NSI source followed by tandem mass spectrometry (MS/MS) in a Thermo Scientific Orbitrap HF coupled online to UPLC. The applied electrospray voltage was 2.0 kV. The m/z scan range was from 350 to 1800 for the full scan. Intact peptides were detected in the Orbitrap at a resolution of 70,000. Peptides were then selected for MS/MS using an NCE setting of 28, and the fragments were detected in the Orbitrap at a resolution of 17,500. A data-dependent procedure alternated between one MS scan followed by 20 MS/MS scans with a 15-s dynamic exclusion. The automatic gain control was set at 5E4. The resulting MS/MS data were processed using the MaxQuant software. Tandem mass spectra were searched for UniProt *B. vallismortis*. Trypsin/P was used as the cleavage enzyme. Mass error was set to 10 ppm for precursor ions and 0.02 Da for fragment ions. Carbamidomethyl on Cys was specified as a fixed modification, and oxidation on Met and Acetyl (protein N-term) were specified as variable modifications.

### 2.8. Heterologous Expression of Potential Acaricidal Proteins from NBIF-001

The six candidate protein genes (B9C48_02315, B9C48_05580, B9C48_07375, B9C48_07725, B9C48_18385, and B9C48_18675) were amplified from the total DNA of NBIF-001 using the designed primers (Table S1) and ExTaq polymerase (TransGen Biotech, Beijing, China). The cloned fragments were inserted into multiple cloning sites of the vector pET28a. The recombinant vectors were transformed into *E. coli* 5α, which was confirmed by sequencing using the universal primers T7 and T7ter. The plasmids were extracted and transformed into *E. coli* BL21 (DE3) cells. The inducible expression of the six His-tagged proteins was conducted according to a method described by [30]. The target proteins were purified using His-Bind columns (Qiagen, Germany), according to the manufacturer’s protocol. Purified proteins were detected by SDS-PAGE, and the protein concentration was calculated using Image Lab 6.1.0 (Bio-Rad, Hercules, CA, USA).

### 2.9. Statistical Analysis

The 50% lethal concentration (LC_50_) values and 95% confidence limits were calculated from three independent experiments using PROBIT software (SPSS Statistics v22.0, IBM). The data analysis was performed using a one-way analysis of variance (ANOVA) with SPSS Statistics v22.0 (IBM). Duncan’s multiple range and Tukey’s HSD tests were used to identify the differences among the experimental groups. Results with a *p*-value < 0.05 were considered statistically significant.

## 3. Results

### 3.1. Isolation of the Strain NBIF-001 That Exhibits Acaricidal Activity against Spider Mites

To screen for beneficial bacterial resources that may control citrus red mites, 475 soil samples from 13 provinces in China were used for strain isolation, and 3343 strains were obtained over the past five years. When using *T. urticae* as targets, isolated NBIF-001 displayed acaricidal activity during the preliminary screening evaluation by slide-dip immersion [22]. The overnight culture supernatant was found to kill *T. urticae* in 24 h. Isolated NBIF-001 was therefore selected to further assess acaricidal activity.

**Figure 1:**
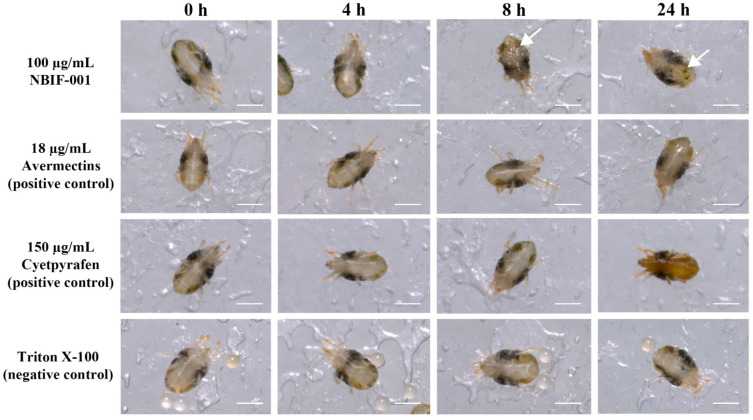
Female adult *Tetranychus urticae* (green) treated with 100.0 µg/mL of NBIF-001 culture re-suspension, 18.0 µg/mL avermectins, 150.0 µg/mL cyetpyrafen (positive controls), and 0.05% Triton X-100 (negative control) for 24 h. Approximately 120 active adult mites were used for each individual treatment. Bars represent 250 μm. Arrows indicate signs of body dehydration.

To precisely measure the mortality rate, the gradient concentrations of the lyophilised powders of the NBIF-001 culture (≥90% spores formed, 3.2 × 10^10^ CFU/g) were used. As shown in Table 1, NBIF-001 exhibited strong acaricidal activity against the three types of spider mites. The 100 µg/mL (3.2 × 10^6^ CFU/mL) treatments killed more than 90% of the spider mites in 48 h. Based on the mortality data, the median LC_50_ was 50.2 µg/mL for *T. urticae* (red form), 18.0 µg/mL for *T. urticae* (green form), and 15.7 µg/mL for *P. citri*. Microscopic observation indicated that after exposure to 100 µg/mL (3.2 × 10^6^ CFU/mL) of NBIF-001 solution for 5 s, the bodies of *T. urticae* were dehydrated within 8 h, while the mites remained alive for 24 h in the solvent control treatment. In the positive control treatments, the mites were killed by 18.0 µg/mL avermectins in 24 h, but their bodies did not show visible change. Furthermore, the mites were killed by 150.0 µg/mL cyetpyrafen in 24 h, in which the bodies remained intact but the colour darkened (Figure 1). This demonstrated that isolated NBIF-001 exhibited excellent acaricidal activity against *T. urticae* (red and green) and *P. citri* in the laboratory.

### 3.2. Raising Fermentation Level Increased Acaricidal Activity

To investigate the field efficacy of NBIF-001 against *P. citri*, fermentation optimisation experiments were performed. In the carbon and nitrogen source experiments, five carbon sources (glucose, glycerol, sucrose, corn flour, and corn starch) were tested. The numbers of cells and spores were the highest with corn flour at 1.1 × 10^10^ CFU/mL and 8.6 × 10^9^ CFU/mL, respectively (Figure S1b). Nine nitrogen sources (urea, fish meal, soybean meal, industrial peptone, yeast extract, silkworm pupa meal, peanut meal, sesame meal, and corn steep liquor) were further tested. The highest numbers of cells and spores were generated with soybean meal at 1.2 × 10^10^ and 9.0 × 10^9^ CFU/mL, respectively (Figure S1c). The other optimal conditions were pH 7.0, speed 210 rpm, 30 °C, and 3% inoculation (Figure S1d–g). In the later stages of the study, the optimal fermentation media for isolated NBIF-001 were corn flour (30.0 g/L), soybean meal (30.0 g/L), yeast extract (15.0 g/L), corn steep liquor (10.0 g/L), calcium carbonate (1.0 g/L), magnesium sulphate (0.3 g/L), and potassium dihydrogen phosphate (0.1 g/L). The spore number reached 1.3 × 10^10^ CFU/mL, which was 2.6-fold the number under the initial conditions (5.0 × 10^9^ CFU/mL). The acaricidal activity assay showed that the optimised culture powder of NBIF-001 (7.6 × 10^10^ CFU/g) exhibited higher acaricidal activity and that 50.0 µg/mL treatments killed all the spider mites (Table 2). Based on the mortality data, the LC_50_ values were 28.8 µg/mL for *T. urticae* (red), 8.3 µg/mL for *T. urticae* (green), and 6.2 µg/mL for *P. citri*, which indicated an approximately two-fold increase. Acaricidal activity therefore increased with increasing fermentation potency.

### 3.3. Field Efficacy of NBIF-001 Wettable Powder against P. citri

The field efficacy experiments showed that 50×, 100×, and 200× dilutions of NBIF-001 exhibited acaricidal activity against *P. citri*. The 50× and 100× dilution treatments both exhibited the highest activity, which was up to 95.8 ± 1.0% and 93.2 ± 2.5% corrected field efficacy after 1 day and remained at 88.3 ± 1.7% and 88.9 ± 5.2% after 7 days, respectively, while the efficacies of the chemical control at routine dosages were 87.5 ± 1.4% after 1 day and 75.1 ± 3.9% after 7 days. The 200× dilution treatment showed 92.0 ± 3.4% efficacy after 1 day, but decreased to 59.9 ± 9.2% in 7 days, which was lower than that of the chemical control (Table 3). This demonstrates that NBIF-001 worked well in the biocontrol of *P. citri*. After 14 days, only the 50× dilution of NBIF-001 showed a slightly lower field efficacy than the chemical control treatment (67.1 ± 7.9% and 71.1 ± 6.4%, respectively). The 100× and 200× dilutions of NBIF-001 showed a significant decrease in field efficacy (59.5 ± 20.0% and 43.3 ± 13.6%, respectively, Table 3). Based on the meteorological conditions shown in Table S2, it rained on the 7th, 9th, and 10th days after treatment. Given that the wettable powder of NBIF-001 used in the field was made in the laboratory, the agents may not have exerted as strong an adhesive effect on the leaves as the commercial cyetpyrafen 30% SC and may have been lost in the rain, which may explain the rapid decline in efficiency.

### 3.4. Genomic Analysis and Special Features Suggesting Putative Acaricidal Compounds

**Figure 2:**
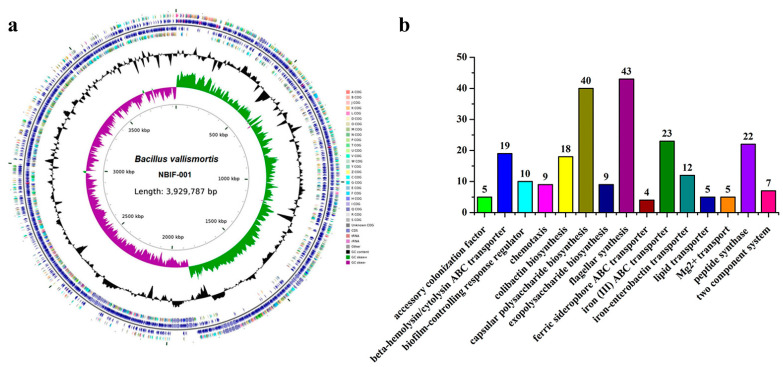
Genomic and acaricidal factors analyses. (**a**) Circular genome map of *Bacillus vallismortis* NBIF-001 using CG View. The outermost and second circles of all replicons indicate the CDS on the forward and reverse strands, which were coloured according to the COG category. The third and fourth circles show the GC content and GC skew in green (+) and purple (−), respectively. The scale is shown in the innermost circle. (**b**) The distribution of functional annotated genes involved in acaricidal activity.

To investigate the acaricidal compounds, the strain NBIF-001 was identified and analysed by genome sequencing. According to the 16S rRNA gene alignment, NBIF-001 belongs to *B. vallismortis* [21]. The genome of *B. vallismortis* NBIF-001 is composed of a circular chromosome of 3,929,787 bp (Figure 2a) with an overall GC content of 46.5%, 3774 protein-coding sequences, 86 tRNA genes, 27 rRNA genes, and 82 other noncoding RNA genes. Since insencticidal factors must be secreted [31], we focused on the genes that encode proteins with signal sequences by utilising SignalP 6.0 [27] and found 325 putative secreted proteins (Table S3). A combined analysis with the Virulence Factors Database [26] showed that 231 acaricidal activity related factors were distributed among 15 catalogues (Figure 2b). These secreted enzymes, hypothetical proteins, and peptide synthases may play key roles in acaricidal activity.

We further used the publicly released *B. vallismortis* DSM 11031 genome as a reference (GenBank accession no. CP026362.1) for an antiSMASH analysis, which revealed that the genomes of *B. vallismortis* NBIF-001 and DSM 11031 contain gene clusters of bacilysin, surfactin, fengycin, and bacillibactin. NBIF-001 contains gene clusters of macrolactin, difficidin, and bacillaene (Table 4), whereas DSM 11031 contains a gene cluster of subtilin. *B. thuringiensis* HD-1 contains only petrobactin and fengycin (Figure S2). These seven different clusters indicate that *B. vallismortis* NBIF-001 may have potential for antimicrobial application. According to a previous study on *B. velezensis* W1 [20], the role of these compounds in acaricidal activity needs to be further researched.

### 3.5. Proteins in the Fermentation Broth Supernatants of NBIF-001 Show Toxicity against Spider Mites

According to the genomics analysis and previous work on the toxins and insecticidal factors in *B. thuringiensis*, which are a successful source of bio-insecticides [31], it was hypothesised that the fermentation broth supernatant of NBIF-001 was acaricidal. To test this hypothesis, bioassays of the broth, supernatants, and cells to show their activity against *T. urticae* were performed. The supernatants exhibited acaricidal activity that was comparable to that of the fermentation broth. The 50× dilution treatments killed 72.5 ± 5.4% of the mites, whereas the broth killed 78.4 ± 4.0%. The cell resuspension had lower toxicity, and the 10× dilution treatments killed 57.0 ± 13.4% of the mites (Table 5). These results indicate that acaricidal compounds were mainly present in the supernatants.

To investigate the acaricidal components of the fermentation broth supernatants, total proteins were crudely extracted using the ammonium sulphate precipitation method. Precipitation was observed in all fractions (30%, 50%, 70%, and 90% ammonium sulphate saturation). The bioassays showed that all the fractions exhibited acaricidal activity. The 50% fraction displayed the highest toxicity, with an LC_50_ of 261.7 μg/mL (Table S4). This indicates that the proteins in the fermentation broth supernatants were toxic to the spider mites.

### 3.6. Proteins of NBIF-001 Are Novel Acaricidal Compound against Spider Mites

A mass spectrometric analysis of the 50% fraction was performed to identify the acaricidal proteins in the fermentation broth supernatants of NBIF-001. The 15 most-abundant proteins in the supernatants are listed in Table S5. Based on the annotation and abundance of the proteins, six were selected for heterologous expression in *E. coli*: peptidase S8 (BV07725), proteinase inhibitor (BV05580), serine hydrolase (BV07375), and three hypothetical proteins (BV02315, BV18385, and BV18675; Table S5). Five of these proteins were successfully purified (Figure S3).

**Figure 3:**
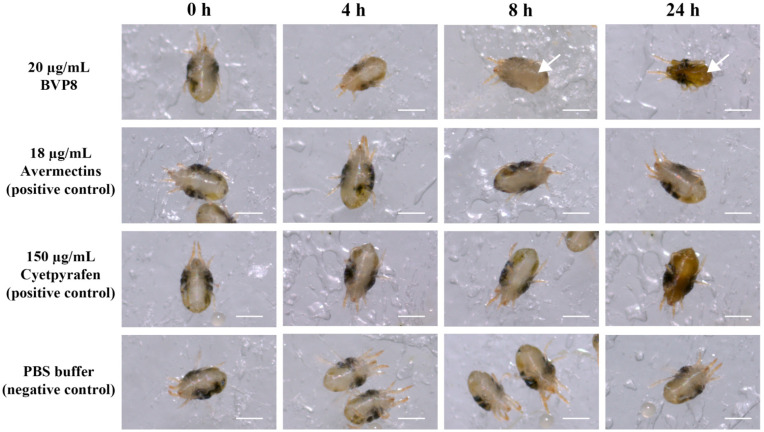
Female adult *Tetranychus urticae* (green) treated with 20.0 µg/mL purified BVP8 proteins, 18.0 µg/mL avermectins, 150.0 µg/mL cyetpyrafen (positive controls), and phosphate-buffed saline buffer (negative control) for 24 h. Approximately 120 active adult mites were used for each individual treatment. Bars represent 250 μm. Arrows indicate signs of body dehydration.

The acaricidal activity of the purified proteins against *T. urticae* was investigated. Compared with the BSA control (1000.0 μg/mL), most of the purified proteins exhibited relatively low acaricidal activity, including the proteinase inhibitor and serine hydrolase (Table 6). BV18385, which is a hypothetical protein that does not contain any known conserved domains, exhibited high toxicity (LC_50_ 12.4 μg/mL) against *T. urticae*. Owing to the particularity of BV18385, this protein was renamed as BVP8 (*B. vallismortis* Protein 8 kDa). Acaricidal phenotypic observations of BVP8 were also made. The photographs showed that after 8 h of treatment with 20.0 μg/mL of BVP8, the mites appeared inactivated and discoloured. After 24 h of treatment, the mites died and had shrunken. The mites under positive control treatments displayed consistent phenotypes, as mentioned above (Figure 3). These results indicate that various NBIF-001 proteins, especially BVP8, are novel acaricidal compound against spider mites.

## 4. Discussion

Hundreds of acaricides have been developed to effectively control spider mites, but many have been removed from the market because mites have evolved high levels of resistance [5,6,32,33,34]. This is due to the frequent spraying by farmers, which occurs approximately 20 times a year [4]. It is also due to the fecundity (more than 100 eggs/female) [35] and short reproductive cycle (approximately 10 days) of the mites [36]. Moreover, spider mites can reproduce through a haploid–diploid system called arrhenotokous. Arrhenotokous reproduction enables female mites to produce unfertilised eggs, which can develop into haploid males. When the mites suffer irritation caused by acaricides during the reproductive cycle, they can overexpress metabolic enzymes to rapidly detoxify acaricides and/or select rare mutant alleles to confer target-site resistance [37,38]. In the arrhenotokous process, unfertilised females first produce only male offspring, followed by bisexual reproduction wherein fertilised females produce both sexes, with a strong preponderance of females. Since the high mutation rate favours the development of resistance alleles, the phenomenon of arrhenotoky leads to a faster fixation of possible resistance alleles, and recessive traits become immediately visible in the population [37,38].

The chemical control of spider mites is currently the preferred approach in orchards, greenhouses, and fields worldwide but has many limitations due to the development of resistance in mites [35]. Propargite, the top acaricide registered in China, is an inhibitor of mitochondrial ATP synthase in mites [39]. Since its introduction in China over 50 years ago, resistance has been frequently reported [40,41]. Glutathione S-transferases have been found to be involved in the resistance to propargite, which has a 37.78-fold resistance ratio [40]. Abamectin, a macrocyclic lactone found in the actinomycete *Streptomyces*, has been used as an insecticide and acaricide for decades [42,43]. Its target site in mites is the glutamate-gated chloride channel, and recently, many point mutations have been reported to confer resistance [5,44]. The newly developed acaricide cyetpyrafen is a mitochondrial electron transport inhibitor of complex II that has been used in China for spider mite control since 2016 [45]. However, resistant strains were observed in 2018 at low levels in cultivated strawberries [34,46].

Given these difficulties, biological control seems to offer solutions to reduce chemical usage and delay resistance. The seasonal release of natural enemies that suppress pest population outbreaks is an important method [47]. Predatory mites (Acari: Phytoseiidae) are used to control phytophagous mites and small insects, including *T. urticae* [47]. To effectively control the mites, commercially available Phytoseiidae should be released in orchards and greenhouses two or three months before and several times during the damage period of spider mites. However, it is still difficult to suppress spider mite densities below the control threshold, which is usually combined with selected chemical acaricides [9]. Essential oils are secondary plant metabolites that are synthesised by plants as a self-defence mechanism and are being developed as botanical acaricides. Natural thyme oil has shown greater toxicity than its single constituent or a blend of constituents in laboratories [48]. The essential oil from *Varronia curassavica* has also exhibited toxicity to *T. urticae* and green aphids (*Myzus persicae* Sulzer) but has not affected the survival of predatory insects, such as green lacewings (*Ceraeochrysa cubana* Hagen) [49].

Microbial agents have mature production systems and have been used for pest control for already for a long time. With increasing legal restrictions on the residue levels of most synthetic chemical pesticides [7], microbial pesticides may be better marketed [50]. However, the development of microbial acaricides for spider mite control is challenging. Owing to the feeding pattern of piercing-sucking insects, it is impossible to make them feed on bacterial cells, spores, and toxins comprising *B. thuringiensis* agents, even though this species has been successfully used in insect biocontrol worldwide [16]. Mycoinsecticides, including *B. bassiana* and *M. anisopliae*, have been tested for acaricidal activities against *T. urticae* and have shown some application potential [11,12]. Surfactants and hydrolytic enzymes, such as proteases and glucanases identified from *B. atrophaeus*, have been reported to cause a 59.8% mortality rate in aphids by affecting cuticle membranes [51]. Cyclodipeptides [19], bacillomycins, macrolactins, and surfactins [20] identified in *B. velezensis* W1 may also have acaricidal potential. However, to the best of our knowledge, no research has been published on microbial acaricides that may be used in fields.

In this study, we identified the *B. vallismortis* strain NBIF-001, which has the capacity to kill the spider mites *T. urticae* (green and red form) and *P. citri*. The signs of biological and chemical treatments on the dead bodies were significantly different, which implies that NBIF-001 and its protein BVP8 have different mechanisms of action or target sites from those of the chemical acaricides used in the bioassays. The different LC_50_ for different taxa of spider mites, especially the results between green and red forms of *T. urticae*, might be partially due to the systematical errors among these independent bioassays since the values were at a similar and comparable level. Our experiments demonstrated that proteins from NBIF-001 supernatants, especially the small protein BVP8, exert acaricidal activity. BVP8 is 76 aa in length and is annotated as a hypothetical protein without a conserved functional domain. Structural prediction showed that it has alpha helices (by the SWISS Model, data not shown due to a low identity at 23.7%) and may be a pore-forming toxin. According to the well-researched infection mode of *B. thuringiensis* against insects [16,31], insecticidal proteins cannot usually maintain their function and structure following long-term exposure to sunlight (ultraviolet rays) and at high temperatures in fields or on plant leaves. Thus, the fast action time of BVP8 treatment on spider mites suggests that it has good potential for commercialisation. Studies on the delivery and killing mechanisms of BVP8 in spider mites and their stabilisation may also contribute to the development of novel microbial acaricides. Since the proteins were found to be acaricidal factors, further studies would be performed to clarify the relationship between the extracellular proteins production and acaricidal activity by cell cultivation optimization combined with bioassays. Additionally, the expression and purification of the peptidase family protein BV07725, the first ranking protein in the MS analysis (Supplementary Table S5), is in progress due to its large size (1431 aa in length) and molecular weight (154.4 kDa). Further investigations may reveal whether secreted enzymes are also involved in the toxicity. 

During the field experiments, the data obtained from the 14-day efficacy study suggested that high dilutions of NBIF-001 lab-made powder, with fewer cells and metabolites, had weaker adhesive properties than commercial cyetpyrafen 30% SC after it had rained. This may partially explain the decrease in field efficacy of both the biological agents and chemical controls. Therefore, the application measures and colonisation capability of bacteria on the leaves significantly influenced the effects of the biocontrol. This suggests that, in addition to further studies on the killing mechanisms, a dosage form study may be important to improve the field efficacy of *Bacillus* acaricides.

## 5. Conclusions

In conclusion, *Bacillus* is a potential biocontrol resource that could play an important role in the sustainable development of global agriculture. *B. vallismortis* NBIF-001 exhibited excellent acaricidal activity both indoors and in the field. This study identified a protein factor that killed spider mites and proposes that the control efficacy of *Bacillus* products is equivalent to that of chemical acaricides in the field. Further studies on NBIF-001 and its acaricidal proteins may therefore facilitate the development of novel microbial acaricides.

## Figures and Tables

**Table 1:** Acaricidal activity of *Bacillus vallismortis* NBIF-001 and *B. thuringiensis* HD-1 (control) against *Tetranychus urticae* (green and red) and *Panonychus citri*.

Strains	Concentration (μg/mL)	Concentration (CFU/mL)	Corrected Mortality (%) and LC_50_ (95% Confidence Interval) ^a^ (μg/mL)					
			*T. urticae* (green)		*T. urticae* (red)		*P. citri*	
NBIF-001	1.0	3.2 × 10^4^	6.5 ± 3.3	50.2 (45.6–55.4)	16.6 ± 5.3	18.0 (15.2–20.8)	13.2 ± 4.5	15.7 (3.2–28.6)
	10.0	3.2 × 10^5^	10.2 ± 3.8		39.1 ± 7.9		45.1 ± 5.4	
	25.0	8.1 × 10^5^	22.9 ± 2.8		60.1 ± 7.5		71.3 ± 4.4	
	50.0	1.6 × 10^6^	46.9 ± 4.2		96.4 ± 2.5		96.9 ± 3.1	
	100.0	3.2 × 10^6^	94.5 ± 4.8		99.1 ± 1.4		98.0 ± 1.7	
	200.0	6.4 × 10^6^	100.0 ± 0.0		96.6 ± 5.9		100.0 ± 0.0	
HD-1 (negative control)	200.0	-	3.0 ± 3.6	-	1.1 ± 3.3	-	1.8 ± 6.7	-
Avermectins (positive control)	18.0	-	91.9 ± 5.0	-	-	-	-	-
Cyetpyrafen (positive control)	150.0	-	98.9 ± 1.6	-	-	-	-	-

^a^: LC_50_ was determined by a PROBIT analysis; 95% confidence intervals were added.

**Table 2:** Acaricidal activity of fermentation-optimised *Bacillus vallismortis* NBIF-001 against *Tetranychus urticae* (green and red) and *Panonychus citri*.

Concentration (μg/mL)	Concentration (CFU/mL)	Corrected Mortality (%) and LC_50_ (95% Confidence Interval) ^a^ (μg/mL)					
		*T. urticae* (green)		*T. urticae* (red)		*P. citri*	
0.1	7.5 × 10^3^	4.8 ± 2.9	28.8 (21.8–39.0)	13.9 ± 7.2	8.3 (6.8–10.4)	10.0 ± 6.2	6.2 (5.0–7.6)
1.0	7.5 × 10^4^	12.0 ± 4.8		22.0 ± 9.1		26.3 ± 5.3	
10.0	7.5 × 10^5^	26.6 ± 6.2		57.6 ± 15.3		70.4 ± 7.9	
25.0	1.9 × 10^6^	39.8 ± 3.1		78.4 ± 1.5		95.4 ± 2.7	
50.0	3.7 × 10^6^	83.2 ± 7.9		97.0 ± 1.5		97.3 ± 2.7	
100.0	7.5 × 10^6^	99.0 ± 1.4		100.0 ± 0.0		100.0 ± 0.0	
Avermectins (18.0, positive control)	-	91.6 ± 10.9	-	-	-	-	-
Cyetpyrafen (150.0, positive control)	-	100.0 ± 0.0	-	-	-	-	-

^a^: LC_50_ was determined by a PROBIT analysis; 95% confidence intervals were added.

**Table 3:** The corrected field efficacy of *Bacillus vallismortis* NBIF-001 against *Panonychus citri*.

Treatments	1 Day after Treatment		3 Days after Treatment		7 Days after Treatment		14 Days after Treatment	
	Mites Reduced Rate (%)	Corrected Field Efficacy (%)	Mites Reduced Rate (%)	Corrected Field Efficacy (%)	Mites Reduced Rate (%)	Corrected Field Efficacy (%)	Mites Reduced Rate (%)	Corrected Field Efficacy (%)
NBIF-001 50× dilution	94.4 ± 1.6 ^a^	95.8 ± 1.0 ^a^	88.3 ± 4.4 ^a^	91.9 ± 3.2 ^a^	82.0 ± 1.3 ^a^	88.3 ± 1.7 ^a^	65.9 ± 6.4 ^a^	67.1 ± 7.9 ^a^
NBIF-001 100× dilution	90.7 ± 4.0 ^a^	93.2 ± 2.5 ^ab^	84.5 ± 6.3 ^ab^	88.9 ± 5.2 ^a^	83.6 ± 6.7 ^ab^	88.9 ± 5.2 ^a^	61.3 ± 14.4 ^a^	59.5 ± 20.0 ^a^
NBIF-001 200× dilution	89.0 ± 5.3 ^a^	92.0 ± 3.4 ^ab^	61.1 ± 3.5 ^b^	73.1 ± 3.3 ^b^	38.3 ± 9.7 ^b^	59.9 ± 9.2 ^b^	42.2 ± 3.8 ^a^	43.3 ± 13.6 ^a^
Cyetpyrafen 30% SC 2000× dilution	83.1 ± 2.6 ^a^	87.5 ± 1.4 ^b^	75.6 ± 5.8 ^ab^	83.0 ± 4.8 ^ab^	61.5 ± 4.4 ^ab^	75.1 ± 3.9 ^ab^	69.7 ± 6.4 ^a^	71.1 ± 6.4 ^a^
Water control	–34.3 ± 8.1 ^b^	N/A	–46.0 ± 14.8 ^c^	N/A	–57.1 ± 22.1 ^c^	N/A	–7.0 ± 23.1 ^b^	N/A

Note: Different letters indicate significant differences at *p* < 0.05. Treatments with the same letter within each period are not significantly different.

**Table 4:** Antibiotic-related genes in the NBIF-001 genome by antiSMASH.

Genes in NBIF-001	Gene Names	Bacteriocins	Similarity
B9C48_01805-B9C48_01910	*yciC*, *yx01*, *yckC*-*E*, *nin*, *nuc*, *hxlB*, *hxlA*, *hxlR*, *xy02*, *srfAA*, *srfAB*, *comS*, *srfAC*, *srfAD*, *aat*, *ycxC*, *ycxD*, *sfp*, *yczE*, *yckI*, *yckJ*	Surfactin	86%
B9C48_07335-B9C48_07380	*pdhA*, *pks2A*-*pks2I*	Macrolactin	100%
B9C48_08635-B9C48_08700	*acpK*, *baeB*-*baeS*	Bacillaene	100%
B9C48_09260-B9C48_09440	*yngE*-*yngL*, *fenA*-*fenE*, *dacC*	Fengycin	100%
B9C48_11155-B9C48_11225	*difA*-*difO*	Difficidin	100%
B9C48_14735-B9C48_14955	*essA-essC, entC*	Bacillibactin	100%
B9C48_17850-B9C48_17880	*ywfA*, *bacA*-*bacE*, *ywfG*	Bacilysin	100%

**Table 5:** Acaricidal activity of fermentation broth and supernatants of *Bacillus vallismortis* NBIF-001 against *Tetranychus urticae* (green).

Treatments	Dilutions	Corrected Mortality (%)
Fermentation broth	1×	100.0 ± 0.0
	10×	96.3 ± 2.6
	50×	78.4 ± 4.0
	100×	67.4 ± 7.8
	500×	28.1 ± 4.0
	1000×	11.9 ± 2.7
Supernatants	1×	100.0 ± 0.0
	10×	90.5 ± 1.1
	50×	72.5 ± 5.4
	100×	62.7 ± 4.5
	500×	21.7 ± 3.1
	1000×	7.1 ± 5.9
Cell resuspension	1×	90.1 ± 4.8
	10×	57.0 ± 13.4
	50×	25.3 ± 5.0
	100×	10.3 ± 4.3
	500×	1.3 ± 5.9
	1000×	2.3 ± 3.3
Avermectins (positive control)	1000×	92.2 ± 1.3
Cyetpyrafen (positive control)	2000×	100.0 ± 0.0

**Table 6:** Acaricidal activity of purified proteins of *Bacillus vallismortis* NBIF-001 against *Tetranychus urticae* (green).

Proteins	Accession No.	Description	Concentration (μg/mL)	Corrected Mortality (%)	LC_50_ (95% Confidence Interval) ^a^ (μg/mL)
BV02315	ARM26736.1	hypothetical protein	276.4	30.9 ± 15.9	425.1 (327.3–678.5)
			138.2	26.8 ± 6.7	
			69.1	15.3 ± 13.0	
			34.6	8.0 ± 1.5	
BV05580	ARM27309.1	proteinase inhibitor	366.0	50.9 ± 9.7	361.2 (325.5–408.0)
			183.0	33.4 ± 12.3	
			91.5	5.9 ± 2.0	
			45.8	2.1 ± 0.8	
BV07375	ARM27646.1	serine hydrolase	225.6	44.4 ± 8.4	247.0 (207.3–329.8)
			112.8	28.4 ± 3.2	
			56.4	13.3 ± 4.2	
			28.2	4.0 ± 3.0	
BV18385 (BVP8)	ARM29686.1	hypothetical protein	26.9	95.5 ± 4.4	12.4 (10.9–13.9)
			13.4	52.8 ± 6.6	
			6.7	26.7 ± 5.7	
			3.4	15.5 ± 2.5	
BV18675	ARM29738.1	hypothetical protein	262.0	38.3 ± 1.9	302.0 (268.1–354.7)
			131.0	11.4 ± 2.9	
			65.5	3.6 ± 1.1	
			32.7	1.9 ± 1.8	
Avermectins (positive control)	-	-	18.0	91.6 ± 1.9	-
Cyetpyrafen (positive control)	-	-	150.0	100.0 ± 0.0	-

^a^: LC_50_ was determined by a PROBIT analysis; 95% confidence intervals were added.

## Data Availability

Not applicable.

## Supplementary Materials

The following supporting information can be downloaded at: https://www.mdpi.com/article/10.3390/microorganisms10091750/s1, Table S1: Bacterial strains, plasmids and primers used in this study; Table S2: Meteorological conditions on the day of treatment and 14 days after treatment; Table S3: Summary of the predicted secreted proteins of *Bacillus vallismortis* NBIF-001; Table S4: Acaricidal activity of various fractions of precipitated proteins of *Bacillus vallismortis* NBIF-001 against *Tetranychus urticae* Koch; Table S5: Summary of precipitated proteins of *Bacillus vallismortis* NBIF-001; Figure S1: Fermentation optimization of NBIF-001; Figure S2: Secondary metabolites analysis using the antiSAMASH program; Figure S3: SDS-PAGE gel detection of purified proteins.
