# Supplementary material for: Acaricidal Activity and Field Efficacy Analysis of the Potential Biocontrol Agent Bacillus vallismortis NBIF-001 against Spider Mites
Source: Microorganisms. 2022 Aug 30;10(9):1750. doi: 10.3390/microorganisms10091750 (PMC9504962; doi:10.3390/microorganisms10091750)
Supplement: Supplementary file 1 [file microorganisms-10-01750-s001.zip › microorganisms-1863160-supplementary.pdf]

Supplementary material

1 Supplementary Tables

Table S1. Bacterial strains, plasmids and primers used in this study.

Materials	Features
**Strains**	
Bacillus vallismortis NBIF-001	Wild-type, CCTCC M 2015087
B. thuringiensis subsp. *kurstaki* HD-1	Wild-type, BGSC 4D1
Escherichia coli DH5α	F^- φ80lacZΔ M15 Δ (*lacZYA-argF*) *U169 recA1 endA1 hsdR17* (rK^- mK^+) *phoA supE44* λ- *thi–1 gyrA96 relA1*
E. coli BL21 (DE3)	F^- *ompT hsdS*$_B$(r_B^- m_B^-) *gal dcm* (DE3)
**Plasmids**	
pET-28a(+)	P_{T7}, pBR322 ori, Km^R
pET28a-B9C48_02315	PT7::B9C48_02315, pBR322 ori, Km^R
pET28a-B9C48_05580	PT7::B9C48_05580, pBR322 ori, Km^R
pET28a-B9C48_07375	PT7::B9C48_07375, pBR322 ori, Km^R
pET28a-B9C48_07725	PT7::B9C48_07725, pBR322 ori, Km^R
pET28a-B9C48_18385	PT7::B9C48_18385, pBR322 ori, Km^R
pET28a-B9C48_18675	PT7::B9C48_18675, pBR322 ori, Km^R
**Primers***	
02315F	5’-cgcGGATCCATGAAGAAGAAAGTAATTCCTGT-3’
02315R	5’-ccgCTCGAGTTAAATCAAAGCAACAATGAATT-3’
05580F	5’-cgcGGATCCATGAAGTGGCTGTTTGGCCTGTT-3’
05580R	5’-ccgCTCGAGTTATTTTACTTCAAATGTTTTTA-3’
07375F	5’-cgcGGATCCATGAAACAAACAATAAGCAATCC-3’
07375R	5’-ccgCTCGAGTTATTCAATACCTGCCCACACGA-3’
07725F	5’-cgcCATATGTTGAAGAAAAAAACGAGAAACAG-3’
07725R	5’-ccgCTCGAGTTACTCATTGATATAAAGTTTTC-3’
18385F	5’-cgcGGATCCTTGTTCGGACCAATTTTAAA-3’
18385R	5’-ccgCTCGAGTTAGCCAAGAATCATTTTAATAA-3’
18675F	5’-cgcGGATCCATGAACCCATATCAGTACTACAC-3’
18675R	5’-ccgCTCGAGTCAGAAGCCGCCTTCTGT-3’

Km^R: kanamycin resistance.
protective base (lowercase letters) and restriction endonuclease recognition sites (uppercase letters): cgcGGATCC: *Bam*HI; cgcCATATG: *Nde*I; ccgCTCGAG: *Xho*I.

Table S2. Meteorological conditions on the day of treatment and 14 days after treatment.

Date	Maximum Temperature (°C)	Minimum Temperature (°C)	Weather
2022-04-05	23	11	cloudy to clear
2022-04-06	27	12	cloudy
2022-04-07	30	15	clear to cloudy
2022-04-08	31	17	clear to cloudy
2022-04-09	32	16	cloudy to clear
2022-04-10	32	18	cloudy to clear
2022-04-11	33	19	cloudy to clear
2022-04-12	32	15	cloudy to rain
2022-04-13	17	11	cloudy
2022-04-14	19	13	rain
2022-04-15	14	12	rain
2022-04-16	14	10	cloudy
2022-04-17	18	11	cloudy
2022-04-18	22	12	cloudy
2022-04-19	24	12	cloudy to clear

Table S3. Summary of the predicted secreted proteins of *Bacillus vallismortis* NBIF-001.

Protein ID	SignalP type	Protein length (aa)	Description
ARM26351.1	SP(Sec/SPI)	443	D-alanyl-D-alanine carboxypeptidase
ARM26382.1	SP(Sec/SPI)	404	hypothetical protein
ARM26411.1	SP(Sec/SPI)	639	cell division protein FtsH
ARM26414.1	SP(Sec/SPI)	296	peptidylprolyl isomerase
ARM26440.1	SP(Sec/SPI)	49	50S ribosomal protein L33
ARM26476.1	SP(Sec/SPI)	431	preprotein translocase subunit SecY
ARM26496.1	LIPO(Sec/SPII)	185	spore gernimation protein GerD
ARM29881.1	SP(Sec/SPI)	336	iron ABC transporter permease
ARM26507.1	LIPO(Sec/SPII)	318	iron-uptake system-binding protein
ARM26509.1	SP(Sec/SPI)	414	hypothetical protein
ARM26510.1	SP(Sec/SPI)	637	hypothetical protein
ARM26511.1	SP(Sec/SPI)	427	penicillin binding protein PBP4B
ARM26519.1	SP(Sec/SPI)	479	YbbR-like domain-containing protein YbbR
ARM26540.1	SP(Sec/SPI)	158	DUF4879 domain-containing protein
ARM26544.1	SP(Sec/SPI)	246	hypothetical protein
ARM26545.1	SP(Sec/SPI)	402	hypothetical protein
ARM26547.1	SP(Sec/SPI)	295	glycerophosphodiester phosphodiesterase
ARM26582.1	TAT(Tat/SPI)	583	alkaline phosphatase
ARM26583.1	SP(Sec/SPI)	65	twin-arginine translocase TatA/TatE family subunit
ARM26584.1	LIPO(Sec/SPII)	241	twin-arginine translocase subunit TatC
ARM26586.1	SP(Sec/SPI)	375	aminotransferase
ARM26589.1	LIPO(Sec/SPII)	371	L-asparaginase
ARM26590.1	SP(Sec/SPI)	214	esterase
ARM26592.1	LIPO(Sec/SPII)	331	DUF4352 domain-containing protein
ARM26595.1	SP(Sec/SPI)	167	peptidase M15
ARM29886.1	LIPO(Sec/SPII)	319	zinc ABC transporter substrate-binding protein
ARM26611.1	LIPO(Sec/SPII)	293	glycine/betaine ABC transporter
ARM26618.1	SP(Sec/SPI)	209	amino acid transporter
ARM26623.1	SP(Sec/SPI)	94	antimicrobial peptide, Lci
ARM26643.1	SP(Sec/SPI)	107	hypothetical protein
ARM26646.1	SP(Sec/SPI)	145	sporulation protein
ARM26669.1	LIPO(Sec/SPII)	408	spore gernimation protein KC
ARM26673.1	LIPO(Sec/SPII)	263	amino acid ABC transporter substrate-binding protein
ARM26679.1	SP(Sec/SPI)	476	macrolide ABC transporter permease
ARM26683.1	LIPO(Sec/SPII)	39	phosphatase
ARM26686.1	LIPO(Sec/SPII)	317	iron ABC transporter permease
ARM26689.1	LIPO(Sec/SPII)	314	ABC transporter
ARM26699.1	SP(Sec/SPI)	203	hypothetical protein
ARM29888.1	SP(Sec/SPI)	542	copper transporter
ARM26714.1	SP(Sec/SPI)	81	hypothetical protein
ARM26716.1	LIPO(Sec/SPII)	667	penicillin-binding protein

ARM26727.1	LIPO(Sec/SPII)	361	lipoprotein YdaJ
ARM26729.1	SP(Sec/SPI)	575	hypothetical protein
ARM26731.1	SP(Sec/SPI)	701	hypothetical protein
ARM26736.1	SP(Sec/SPI)	149	hypothetical protein
ARM29891.1	SP(Sec/SPI)	106	hypothetical protein
ARM26770.1	LIPO(Sec/SPII)	335	DUF4367 domain-containing protein
ARM26821.1	SP(Sec/SPI)	127	hypothetical protein
ARM26840.1	SP(Sec/SPI)	717	hypothetical protein
ARM26858.1	SP(Sec/SPI)	84	hypothetical protein
ARM26861.1	LIPO(Sec/SPII)	188	hypothetical protein
ARM26884.1	LIPO(Sec/SPII)	63	hypothetical protein
ARM26890.1	LIPO(Sec/SPII)	675	penicillin-binding protein
ARM26895.1	SP(Sec/SPI)	105	hypothetical protein
ARM26898.1	SP(Sec/SPI)	252	hypothetical protein
ARM26904.1	SP(Sec/SPI)	122	hypothetical protein
ARM26905.1	SP(Sec/SPI)	348	hypothetical protein
ARM26931.1	LIPO(Sec/SPII)	331	lipoprotein YerB
ARM26937.1	LIPO(Sec/SPII)	398	hypothetical protein
ARM26945.1	SP(Sec/SPI)	1047	Swarming motility protein SwrC
ARM26957.1	SP(Sec/SPI)	86	spore coat protein
ARM26977.1	LIPO(Sec/SPII)	322	sugar dehydratase
ARM26995.1	SP(Sec/SPI)	421	pectate lyase
ARM27002.1	SP(Sec/SPI)	320	hypothetical protein
ARM27012.1	LIPO(Sec/SPII)	147	hypothetical protein
ARM27023.1	SP(Sec/SPI)	379	yibE/F-like family protein
ARM29903.1	SP(Sec/SPI)	1419	2',3'-cyclic-nucleotide 2'-phosphodiesterase
ARM27035.1	SP(Sec/SPI)	263	hypothetical protein
ARM27039.1	SP(Sec/SPI)	263	delta-lactam-biosynthetic de-N-acetylase
ARM27069.1	SP(Sec/SPI)	362	acyltransferase
ARM27082.1	LIPO(Sec/SPII)	129	hypothetical protein
ARM27083.1	SP(Sec/SPI)	303	serine protease
ARM27086.1	SP(Sec/SPI)	175	hypothetical protein
ARM27090.1	SP(Sec/SPI)	861	hypothetical protein
ARM27109.1	LIPO(Sec/SPII)	573	ABC transporter substrate-binding protein
ARM27117.1	LIPO(Sec/SPII)	329	sulfonate ABC transporter substrate-binding protein
ARM27121.1	LIPO(Sec/SPII)	155	hypothetical protein
ARM27136.1	SP(Sec/SPI)	123	hypothetical protein
ARM27137.1	SP(Sec/SPI)	315	ABC transporter permease
ARM27139.1	LIPO(Sec/SPII)	276	hypothetical protein
ARM27142.1	SP(Sec/SPI)	223	hypothetical protein
ARM27143.1	LIPO(Sec/SPII)	179	lipoprotein YhcN
ARM29909.1	SP(Sec/SPI)	300	hypothetical protein
ARM27145.1	SP(Sec/SPI)	684	bifunctional metallophosphatase/5'-nucleotidase
ARM27146.1	LIPO(Sec/SPII)	198	class D sortase
ARM27161.1	SP(Sec/SPI)	107	hypothetical protein

ARM27162.1	SP(Sec/SPI)	484	peptidoglycan endopeptidase
ARM27167.1	SP(Sec/SPI)	459	alkaline phosphatase
ARM27168.1	SP(Sec/SPI)	274	peptidoglycan endopeptidase
ARM27187.1	SP(Sec/SPI)	282	polysaccharide deacetylase
ARM27207.1	SP(Sec/SPI)	117	hypothetical protein
ARM27216.1	LIPO(Sec/SPII)	285	peptidylprolyl isomerase
ARM27223.1	TAT(Tat/SPI)	118	hypothetical protein
ARM27248.1	SP(Sec/SPI)	131	hypothetical protein
ARM27252.1	LIPO(Sec/SPII)	292	iron-dicitrate ABC transporter substrate-binding protein
ARM27264.1	LIPO(Sec/SPII)	236	hypothetical protein
ARM27265.1	LIPO(Sec/SPII)	89	hypothetical protein
ARM27271.1	SP(Sec/SPI)	171	signal peptidase I
ARM27303.1	LIPO(Sec/SPII)	325	BMP family ABC transporter substrate-binding protein
ARM27304.1	SP(Sec/SPI)	549	histidine kinase
ARM27309.1	LIPO(Sec/SPII)	153	proteinase inhibitor
ARM27315.1	SP(Sec/SPI)	478	FAD-binding oxidoreductase
ARM27325.1	LIPO(Sec/SPII)	61	DUF2929 domain-containing protein
ARM27328.1	LIPO(Sec/SPII)	317	BMP family ABC transporter substrate-binding protein
ARM29918.1	LIPO(Sec/SPII)	542	peptide-binding protein
ARM27336.1	SP(Sec/SPI)	316	peptide ABC transporter permease
ARM27340.1	LIPO(Sec/SPII)	540	ABC transporter substrate-binding protein
ARM27341.1	SP(Sec/SPI)	311	peptide ABC transporter permease
ARM27345.1	SP(Sec/SPI)	395	GTP-binding protein
ARM29919.1	SP(Sec/SPI)	670	oligoendopeptidase F
ARM27353.1	SP(Sec/SPI)	228	lytic transglycosylase
ARM27379.1	SP(Sec/SPI)	249	BCII family subclass B1 metallo-beta-lactamase
ARM27394.1	SP(Sec/SPI)	304	class A beta-lactamase
ARM27398.1	SP(Sec/SPI)	370	arabinogalactan endo-1,4-beta-galactosidase
ARM27412.1	LIPO(Sec/SPII)	189	hypothetical protein
ARM27436.1	SP(Sec/SPI)	60	hypothetical protein
ARM27491.1	SP(Sec/SPI)	519	MFS transporter
ARM27496.1	LIPO(Sec/SPII)	539	oligopeptide ABC transporter substrate-binding protein
ARM27570.1	SP(Sec/SPI)	207	cell wall hydrolase
ARM27572.1	SP(Sec/SPI)	164	sporulation protein
ARM27577.1	SP(Sec/SPI)	698	PTS glucose transporter subunit IICBA
ARM27584.1	SP(Sec/SPI)	258	hypothetical protein
ARM27593.1	TAT(Tat/SPI)	287	metallophosphoesterase
ARM27619.1	SP(Sec/SPI)	378	efflux transporter periplasmic adaptor subunit
ARM29931.1	LIPO(Sec/SPII)	212	hypothetical protein
ARM27651.1	LIPO(Sec/SPII)	124	hypothetical protein
ARM27652.1	LIPO(Sec/SPII)	266	polysaccharide deacetylase
ARM27660.1	SP(Sec/SPI)	521	peptidase M4

ARM27663.1	SP(Sec/SPI)	205	hypothetical protein
ARM27668.1	LIPO(Sec/SPII)	207	hypothetical protein
ARM27675.1	SP(Sec/SPI)	308	heme A synthase
ARM27677.1	LIPO(Sec/SPII)	356	cytochrome c oxidase subunit II
ARM27691.1	SP(Sec/SPI)	340	hypothetical protein
ARM27740.1	SP(Sec/SPI)	354	anion permease
ARM27750.1	SP(Sec/SPI)	406	bifunctional 4'-phosphopantothenoylcysteine decarboxylase/phosphopantothenoylcysteine synthetase
ARM27764.1	SP(Sec/SPI)	555	hypothetical protein
ARM27811.1	SP(Sec/SPI)	139	flagellar basal body-associated protein FliL
ARM27815.1	SP(Sec/SPI)	217	flagella biosynthesis protein FliZ
ARM27865.1	SP(Sec/SPI)	1039	hypothetical protein
ARM27901.1	SP(Sec/SPI)	144	sporulation protein
ARM27905.1	LIPO(Sec/SPII)	235	hypothetical protein
ARM27913.1	LIPO(Sec/SPII)	91	hypothetical protein
ARM27939.1	SP(Sec/SPI)	143	hypothetical protein
ARM27945.1	LIPO(Sec/SPII)	370	hypothetical protein
ARM27957.1	SP(Sec/SPI)	142	hypothetical protein
ARM27959.1	LIPO(Sec/SPII)	103	cell division suppressor protein YneA
ARM27984.1	SP(Sec/SPI)	117	hypothetical protein
ARM27995.1	SP(Sec/SPI)	542	hypothetical protein
ARM28000.1	SP(Sec/SPI)	499	endoglucanase
ARM28004.1	SP(Sec/SPI)	423	glucuronoxylanase
ARM28005.1	SP(Sec/SPI)	512	arabinoxylan arabinofuranohydrolase
ARM28020.1	LIPO(Sec/SPII)	135	hypothetical protein
ARM28029.1	SP(Sec/SPI)	512	hypothetical protein
ARM28036.1	SP(Sec/SPI)	491	D-alanyl-D-alaninecarboxypeptidase/D-alanyl-D-alanine-endopeptidase
ARM28038.1	SP(Sec/SPI)	180	hypothetical protein
ARM28041.1	SP(Sec/SPI)	588	gamma-glutamyltransferase
ARM28077.1	SP(Sec/SPI)	289	peptidoglycan-binding protein
ARM28098.1	LIPO(Sec/SPII)	196	superoxide dismutase
ARM28099.1	SP(Sec/SPI)	411	peptidoglycan endopeptidase
ARM28121.1	SP(Sec/SPI)	467	peptidase S41
ARM28124.1	LIPO(Sec/SPII)	272	peptidase M15
ARM28144.1	LIPO(Sec/SPII)	383	3-phytase
ARM29947.1	LIPO(Sec/SPII)	118	hypothetical protein
ARM28155.1	LIPO(Sec/SPII)	316	endonuclease
ARM28161.1	SP(Sec/SPI)	187	hypothetical protein
ARM28162.1	LIPO(Sec/SPII)	251	hypothetical protein
ARM28163.1	LIPO(Sec/SPII)	193	cytochrome c oxidase assembly protein
ARM28171.1	SP(Sec/SPI)	202	hypothetical protein
ARM28194.1	SP(Sec/SPI)	87	transcriptional regulator
ARM28196.1	SP(Sec/SPI)	91	hypothetical protein
ARM28222.1	SP(Sec/SPI)	393	aspartate aminotransferase

ARM28223.1	SP(Sec/SPI)	161	hypothetical protein
ARM28237.1	SP(Sec/SPI)	260	sporulation protein YpjB
ARM28241.1	TAT(Tat/SPI)	167	menaquinol-cytochrome C reductase
ARM29951.1	LIPO(Sec/SPII)	244	hypothetical protein
ARM28266.1	SP(Sec/SPI)	345	glycerol-3-phosphate dehydrogenase (NAD(P)(+))
ARM28275.1	SP(Sec/SPI)	290	spore cortex-lytic enzyme
ARM28280.1	SP(Sec/SPI)	262	hypothetical protein
ARM28299.1	SP(Sec/SPI)	383	D-alanyl-D-alanine carboxypeptidase
ARM28310.1	SP(Sec/SPI)	114	hypothetical protein
ARM28314.1	SP(Sec/SPI)	299	hypothetical protein
ARM28325.1	SP(Sec/SPI)	389	D-alanyl-D-alanine carboxypeptidase
ARM28336.1	SP(Sec/SPI)	329	L-asparaginase 1
ARM28386.1	SP(Sec/SPI)	278	OxaA precursor
ARM28392.1	LIPO(Sec/SPII)	255	ABC transporter substrate-binding protein
ARM28408.1	SP(Sec/SPI)	283	3-hydroxybutyryl-CoA dehydrogenase
ARM28411.1	SP(Sec/SPI)	208	N-acetylmuramoyl-L-alanine amidase
ARM28412.1	LIPO(Sec/SPII)	105	hypothetical protein
ARM28434.1	SP(Sec/SPI)	216	stage III sporulation protein AH
ARM28437.1	SP(Sec/SPI)	402	stage III sporulation protein AE
ARM28460.1	SP(Sec/SPI)	194	S26 family signal peptidase
ARM28461.1	LIPO(Sec/SPII)	223	amyloid fiber anchoring/assembly protein TapA
ARM28462.1	SP(Sec/SPI)	109	hypothetical protein
ARM28465.1	SP(Sec/SPI)	104	competence protein ComGE
ARM28471.1	LIPO(Sec/SPII)	433	hypothetical protein
ARM28478.1	LIPO(Sec/SPII)	345	hypothetical protein
ARM28485.1	SP(Sec/SPI)	117	hypothetical protein
ARM28486.1	SP(Sec/SPI)	154	hypothetical protein
ARM28491.1	LIPO(Sec/SPII)	300	phosphate-binding protein
ARM28497.1	SP(Sec/SPI)	100	hypothetical protein
ARM28510.1	SP(Sec/SPI)	120	cytochrome C
ARM28529.1	SP(Sec/SPI)	438	hypothetical protein
ARM28542.1	SP(Sec/SPI)	112	hypothetical protein
ARM28561.1	LIPO(Sec/SPII)	243	hypothetical protein
ARM28562.1	SP(Sec/SPI)	244	chitooligosaccharide deacetylase
ARM28625.1	LIPO(Sec/SPII)	275	amino acid ABC transporter substrate-binding protein
ARM28640.1	SP(Sec/SPI)	520	N-acetylmuramoyl-L-alanine amidase
ARM28647.1	SP(Sec/SPI)	739	protein translocase subunit SecDF
ARM28658.1	SP(Sec/SPI)	169	signaling peptide protein
ARM28659.1	LIPO(Sec/SPII)	197	sporulation protein
ARM28663.1	LIPO(Sec/SPII)	521	L-aspartate oxidase
ARM28666.1	LIPO(Sec/SPII)	285	prephenate dehydratase
ARM28677.1	LIPO(Sec/SPII)	172	rod shape-determining protein MreD
ARM28678.1	SP(Sec/SPI)	287	rod shape-determining protein MreC
ARM28683.1	LIPO(Sec/SPII)	250	prepilin peptidase
ARM28711.1	LIPO(Sec/SPII)	361	sporulation protein

ARM28717.1	SP(Sec/SPI)	586	succinate dehydrogenase flavoprotein subunit
ARM28745.1	LIPO(Sec/SPII)	431	arabinose-binding protein
ARM28751.1	SP(Sec/SPI)	321	arabinan endo-1,5-alpha-L-arabinosidase
ARM28777.1	SP(Sec/SPI)	579	PAS domain-containing sensor histidine kinase
ARM28779.1	SP(Sec/SPI)	312	malate dehydrogenase
ARM28808.1	SP(Sec/SPI)	335	S49 family peptidase
ARM28876.1	LIPO(Sec/SPII)	425	binding protein msmE
ARM28878.1	SP(Sec/SPI)	273	maltose ABC transporter permease
ARM28902.1	LIPO(Sec/SPII)	269	glycerophosphodiester phosphodiesterase
ARM28911.1	LIPO(Sec/SPII)	334	hypothetical protein
ARM28917.1	SP(Sec/SPI)	49	YtzI protein
ARM28918.1	LIPO(Sec/SPII)	147	hypothetical protein
ARM28945.1	SP(Sec/SPI)	106	hypothetical protein
ARM28950.1	SP(Sec/SPI)	661	chemotaxis protein
ARM28951.1	SP(Sec/SPI)	662	chemotaxis protein
ARM29976.1	SP(Sec/SPI)	661	chemotaxis protein
ARM28958.1	SP(Sec/SPI)	225	peptidase
ARM29978.1	SP(Sec/SPI)	622	penicillin-binding protein
ARM28982.1	SP(Sec/SPI)	533	two-component system sensor histidine kinase DcuS
ARM28984.1	LIPO(Sec/SPII)	357	BMP family ABC transporter substrate-binding protein
ARM28987.1	SP(Sec/SPI)	72	hypothetical protein
ARM28997.1	SP(Sec/SPI)	766	histidine kinase
ARM29012.1	SP(Sec/SPI)	157	type VII secretion protein EssA
ARM29033.1	LIPO(Sec/SPII)	213	hypothetical protein
ARM29043.1	SP(Sec/SPI)	111	bacteriocin
ARM29064.1	SP(Sec/SPI)	325	peptidase M23
ARM29076.1	SP(Sec/SPI)	289	ribonuclease
ARM29082.1	LIPO(Sec/SPII)	428	sugar ABC transporter substrate-binding protein
ARM29085.1	SP(Sec/SPI)	196	kinase
ARM29090.1	SP(Sec/SPI)	371	FAD-dependent oxidoreductase
ARM29091.1	SP(Sec/SPI)	244	hypothetical protein
ARM29101.1	LIPO(Sec/SPII)	271	methionine ABC transporter substrate-binding protein
ARM29121.1	SP(Sec/SPI)	346	2-dehydropantoate 2-reductase
ARM29125.1	LIPO(Sec/SPII)	145	hypothetical protein
ARM29137.1	LIPO(Sec/SPII)	370	spore gernimation protein
ARM29141.1	SP(Sec/SPI)	289	DUF4097 domain-containing protein
ARM29148.1	LIPO(Sec/SPII)	314	ABC transporter substrate-binding protein
ARM29161.1	LIPO(Sec/SPII)	312	iron(3+)-hydroxamate-binding protein fhuD
ARM29167.1	LIPO(Sec/SPII)	265	molybdate ABC transporter substrate-binding protein
ARM29172.1	SP(Sec/SPI)	364	ABC transporter permease

ARM29173.1	SP(Sec/SPI)	381	ABC transporter permease
ARM29174.1	SP(Sec/SPI)	161	stress protein
ARM29180.1	SP(Sec/SPI)	266	peptidase M84
ARM29182.1	SP(Sec/SPI)	223	thioredoxin
ARM29194.1	LIPO(Sec/SPII)	270	amino acid ABC transporter substrate-binding protein
ARM29195.1	LIPO(Sec/SPII)	270	amino acid ABC transporter substrate-binding protein
ARM29211.1	SP(Sec/SPI)	467	two-component sensor histidine kinase
ARM29217.1	LIPO(Sec/SPII)	305	osmoprotectant ABC transporter substrate-binding protein
ARM29222.1	LIPO(Sec/SPII)	305	osmoprotectant ABC transporter substrate-binding protein
ARM29238.1	SP(Sec/SPI)	426	LytTR family transcriptional regulator
ARM29248.1	SP(Sec/SPI)	135	hypothetical protein
ARM29270.1	SP(Sec/SPI)	202	UDP-galactose phosphate transferase
ARM29303.1	LIPO(Sec/SPII)	418	sugar ABC transporter substrate-binding protein
ARM29311.1	SP(Sec/SPI)	157	ribonuclease
ARM29319.1	SP(Sec/SPI)	468	peptidase C40
ARM29340.1	SP(Sec/SPI)	1289	hypothetical protein
ARM29352.1	LIPO(Sec/SPII)	396	cell division protein
ARM29354.1	SP(Sec/SPI)	466	peptidase S41
ARM29355.1	SP(Sec/SPI)	468	hypothetical protein
ARM29357.1	LIPO(Sec/SPII)	113	cytochrome C
ARM29383.1	SP(Sec/SPI)	393	LytR family transcriptional regulator
ARM29387.1	SP(Sec/SPI)	226	hypothetical protein
ARM29389.1	SP(Sec/SPI)	446	UDP-glucose 6-dehydrogenase
ARM29393.1	SP(Sec/SPI)	496	N-acetylmuramoyl-L-alanine amidase
ARM29394.1	SP(Sec/SPI)	706	amidase
ARM29395.1	LIPO(Sec/SPII)	105	LytA
ARM29409.1	LIPO(Sec/SPII)	376	spore gernimation protein GerA
ARM29412.1	SP(Sec/SPI)	328	LytR family transcriptional regulator
ARM29415.1	SP(Sec/SPI)	411	bifunctional murein DD-endopeptidase/murein LD-carboxypeptidase
ARM29422.1	LIPO(Sec/SPII)	377	hypothetical protein
ARM29428.1	LIPO(Sec/SPII)	305	D-ribose ABC transporter substrate-binding protein
ARM29429.1	SP(Sec/SPI)	177	cell wall-binding protein
ARM29444.1	SP(Sec/SPI)	72	hypothetical protein
ARM29482.1	SP(Sec/SPI)	140	hypothetical protein
ARM29489.1	SP(Sec/SPI)	213	1,4-beta-xylanase
ARM29499.1	SP(Sec/SPI)	226	hypothetical protein
ARM29500.1	SP(Sec/SPI)	228	hypothetical protein
ARM29501.1	SP(Sec/SPI)	343	stage II sporulation protein D
ARM29503.1	SP(Sec/SPI)	246	hypothetical protein
ARM29523.1	SP(Sec/SPI)	226	stage II sporulation protein R
ARM29543.1	SP(Sec/SPI)	399	cardiolipin synthase

ARM29602.1	SP(Sec/SPI)	288	protein liaG
ARM29615.1	SP(Sec/SPI)	123	DUF423 domain-containing protein
ARM29616.1	SP(Sec/SPI)	439	purine permease
ARM29627.1	SP(Sec/SPI)	803	peptidase S8
ARM29636.1	LIPO(Sec/SPII)	318	cytochrome aa3 quinol oxidase subunit II
ARM29645.1	TAT(Tat/SPI)	418	deferrochelatase/peroxidase EfeB
ARM29656.1	SP(Sec/SPI)	581	peptidase S8
ARM29665.1	SP(Sec/SPI)	392	D-alanyl-lipoteichoic acid biosynthesis protein DltD
ARM29674.1	LIPO(Sec/SPII)	298	iron-hydroxamate ABC transporter substrate-binding protein
ARM29677.1	SP(Sec/SPI)	103	PTS sugar transporter subunit IIB
ARM29682.1	SP(Sec/SPI)	360	beta-mannosidase
ARM29699.1	LIPO(Sec/SPII)	280	polysaccharide deacetylase
ARM29708.1	SP(Sec/SPI)	224	hypothetical protein
ARM29712.1	SP(Sec/SPI)	243	beta-glucanase
ARM29715.1	SP(Sec/SPI)	354	pectate lyase
ARM29719.1	SP(Sec/SPI)	207	hypothetical protein
ARM29724.1	SP(Sec/SPI)	468	endo-alpha-(1->5)-L-arabinanase
ARM29737.1	LIPO(Sec/SPII)	139	hypothetical protein
ARM29739.1	LIPO(Sec/SPII)	320	iron(3+)-hydroxamate-binding protein yxeB
ARM29740.1	SP(Sec/SPI)	115	hypothetical protein
ARM29769.1	SP(Sec/SPI)	415	hypothetical protein
ARM29785.1	SP(Sec/SPI)	184	Pathogenicity island protein
ARM30016.1	SP(Sec/SPI)	249	hypothetical protein
ARM29790.1	LIPO(Sec/SPII)	326	aldo/keto reductase
ARM29796.1	LIPO(Sec/SPII)	211	hypothetical protein
ARM29830.1	SP(Sec/SPI)	473	glycoside hydrolase 68 family protein
ARM29831.1	SP(Sec/SPI)	514	levanase
ARM29874.1	LIPO(Sec/SPII)	259	OxaA precursor

Table S4. Acaricidal activity of various fractions of precipitated proteins of *Bacillus vallismortis* NBIF-001 against *Tetranychus urticae* Koch.

Treatments	Concentration (µg/mL)	Corrected mortality (%)	LC_{50} (µg/mL)
30% fraction	600	32 ± 10	970.3
	300	26 ± 7	
	150	17 ± 9	
	75	8.4 ± 0.8	
50% fraction	600	85 ± 2	261.7
	300	53 ± 6	
	150	38 ± 6	
	75	32 ± 3	
70% fraction	600	64 ± 15	386.2
	300	42 ± 4	
	150	33 ± 5	
	75	33 ± 5	
90% fraction	600	58 ± 2	448.7
	300	44 ± 7	
	150	31 ± 6	
	75	20 ± 9	

Table S5. Summary of precipitated proteins of *Bacillus vallismortis* NBIF-001.

**Protein ID**	**Gene ID**	**Abundance ranking**	**Protein length (aa)**	**Description**
ARM27713.1	B9C48_07725	1	1431	peptidase S8
ARM27912.1	B9C48_08765	2	73	RNA-binding protein Hfq
ARM29686.1	B9C48_18385	3	76	hypothetical protein
ARM26961.1	B9C48_03625	4	189	spore coat protein CotJC
ARM27912.1	B9C48_08765	5	181	spore coat protein
ARM27809.1	B9C48_08230	6	258	flagellar basal-body rod protein FlgF
ARM27458.1	B9C48_06375	7	311	Phage major capsid protein
ARM30018.1	B9C48_19195	8	161	spore coat protein
ARM27646.1	B9C48_07375	9	363	serine hydrolase
ARM26482.1	B9C48_00865	10	131	30S ribosomal protein S11
ARM27309.1	B9C48_05580	11	153	Proteinase inhibitor
ARM29513.1	B9C48_17445	12	244	ATP synthase subunit A
ARM26736.1	B9C48_02315	13	149	hypothetical protein
ARM27784.1	B9C48_08105	14	115	50S ribosomal protein L19
ARM29738.1	B9C48_18675	15	128	hypothetical protein

2 Supplementary figures

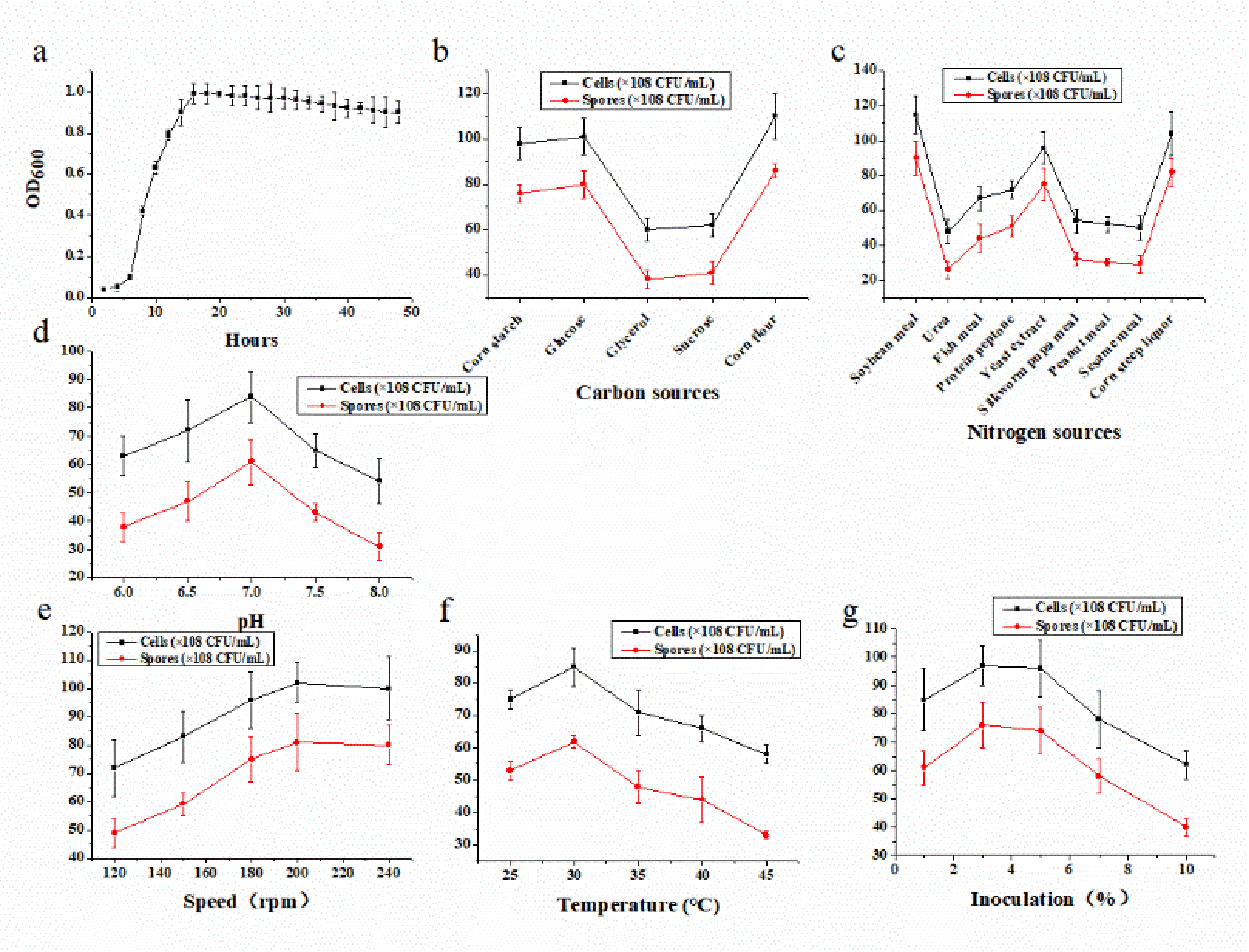

Figure S1. Fermentation optimization of NBIF-001. (a) The growth curve of *B. vallismortis* NBIF-001 in LB medium. (b) Effect of five carbon sources on cells and spores number. (c) Effect of nine nitrogen sources on cells and spores number. (d) Effect of pH on cells and spores number. (e) Effect of rotation speed on cells and spores number. (f) Effect of temperature on cells and spores number. (g) Effect of inoculum rate on cells and spores number.

a

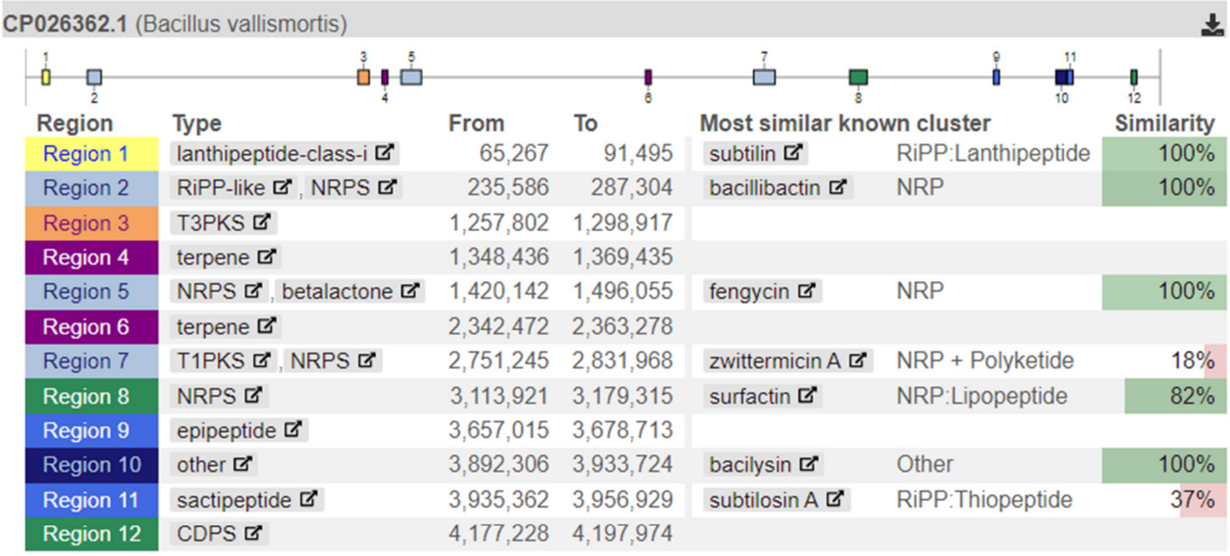

CP026362.1 (Bacillus vallismortis)

Region	Type	From	To	Most similar known cluster		Similarity
Region 1	lanthipeptide-class-i	65,267	91,495	subtilin	RiPP:Lanthipeptide	100%
Region 2	RiPP-like , NRPS	235,586	287,304	bacillibactin	NRP	100%
Region 3	T3PKS	1,257,802	1,298,917			
Region 4	terpene	1,348,436	1,369,435			
Region 5	NRPS , betalactone	1,420,142	1,496,055	fengycin	NRP	100%
Region 6	terpene	2,342,472	2,363,278			
Region 7	T1PKS , NRPS	2,751,245	2,831,968	zwittermicin A	NRP + Polyketide	18%
Region 8	NRPS	3,113,921	3,179,315	surfactin	NRP:Lipopeptide	82%
Region 9	epipeptide	3,657,015	3,678,713			
Region 10	other	3,892,306	3,933,724	bacilysin	Other	100%
Region 11	sactipeptide	3,935,362	3,956,929	subtilosin A	RiPP:Thiopeptide	37%
Region 12	CDPS	4,177,228	4,197,974			

b

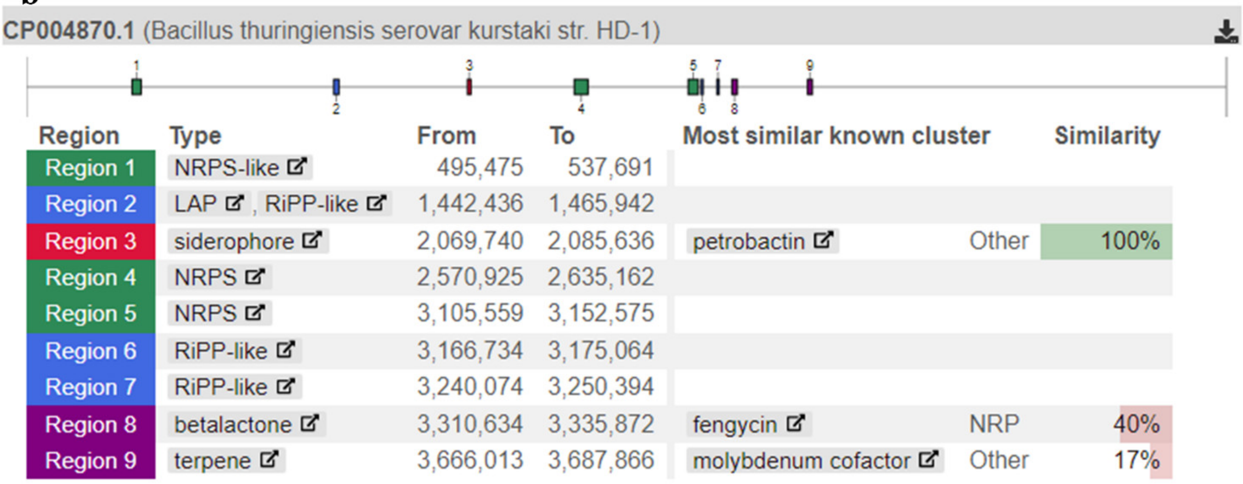

CP004870.1 (Bacillus thuringiensis serovar kurstaki str. HD-1)

Region	Type	From	To	Most similar known cluster		Similarity
Region 1	NRPS-like	495,475	537,691			
Region 2	LAP , RiPP-like	1,442,436	1,465,942			
Region 3	siderophore	2,069,740	2,085,636	petrobactin	Other	100%
Region 4	NRPS	2,570,925	2,635,162			
Region 5	NRPS	3,105,559	3,152,575			
Region 6	RiPP-like	3,166,734	3,175,064			
Region 7	RiPP-like	3,240,074	3,250,394			
Region 8	betalactone	3,310,634	3,335,872	fengycin	NRP	40%
Region 9	terpene	3,666,013	3,687,866	molybdenum cofactor	Other	17%

Figure S2. Secondary metabolites analysis using the antiSAMASH program. (a) *B. vallismortis* DSM 11031. (b) *B. thuringiensis* HD-1.

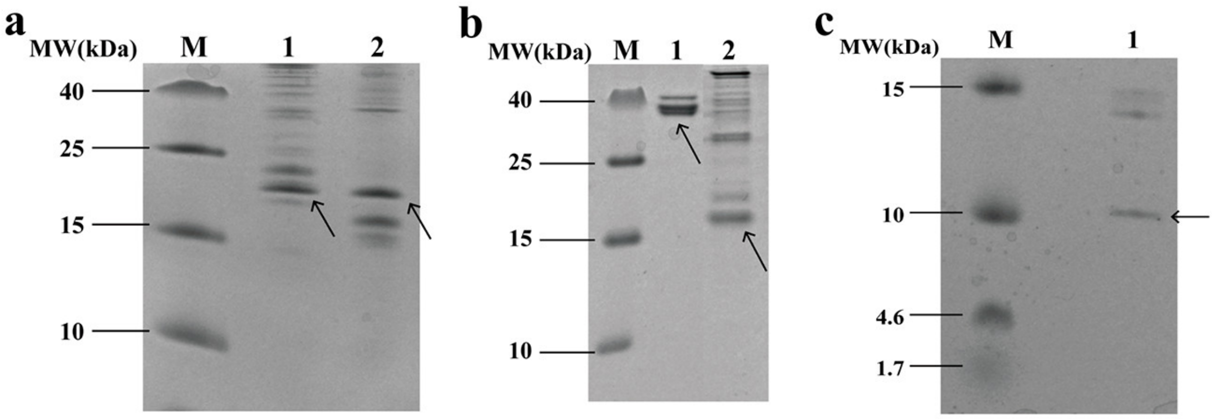

Figure S3. SDS-PAGE gel detection of purified proteins. (a) The purified BV02315 protein (Line 1, 16 kDa) and BV05580 protein (Line 2, 17 kDa) were analyzed using SDS-PAGE and stained with Coomassie blue. (b) The purified BV07375 protein (Line 1, 41 kDa) and BV18675 protein (Line 2, 16 kDa) were analyzed using SDS-PAGE and stained with Coomassie blue. (c) The purified BV18385 protein (Line 1, 8 kDa) was analyzed using SDS-PAGE and stained with Coomassie blue. All Lane M in the figure represent protein marker (26628, Thermos Scientific) and the target proteins were arrowed, respectively.
